# Proteolytic Activity Present in House-Dust-Mite Extracts Degrades ENA-78/CXCL5 and Reduces Neutrophil Migration

**DOI:** 10.1155/2014/673673

**Published:** 2014-05-04

**Authors:** Laura Keglowich, Michael Tamm, Jun Zhong, Nicola Miglino, Pieter Borger

**Affiliations:** ^1^Department of Biomedicine, University Hospital Basel, Hebelstrasse 20, 4031 Basel, Switzerland; ^2^Department of Pulmonology, University Hospital Basel, Petersgraben 2, 4031 Basel, Switzerland

## Abstract

*Background.* Bronchial smooth muscle cells (BSMC) are a major source of proinflammatory and proangiogenic cytokines and chemokines, including VEGF and CXC-chemokines. CXC-chemokines act primarily on neutrophils, mediating their recruitment to and activation at the site of inflammation. In humans, house-dust mite (HDM) allergens can cause asthmatic exacerbations and trigger an inflammatory response through protease-dependent mechanisms. *Objective.* We investigated the effect HDM extract on the release of pro-angiogenic and proinflammatory cytokines from BSMC. *Methods.* Human primary BSMC were stimulated with HDM extract in the absence or presence of fetal calf serum (FCS). Twenty angiogenic cytokines were detected by a specific antibody array and modified protein levels were confirmed by ELISA. Neutrophil migration was measured using a 96-well Boyden chamber. *Results.* ENA-78/CXCL5 protein levels in conditioned medium of BSMC stimulated with HDM extract were significantly reduced (*n* = 10, *P* < 0.05) but restored in the presence of 5% FCS. HDM extracts did not affect ENA-78/CXCL5 mRNA levels. Recombinant ENA-78/CXCL5 was degraded after incubation with HDM extracts (*n* = 7, *P* < 0.05) but restored after the addition of the serine protease AEBSF. Neutrophil migration towards recombinant ENA-78/CXCL5 was also reduced in the presence of HDM extract. *Conclusion.* HDM proteases degrade ENA-78/CXCL5. Thus exposure to HDM allergens may alter ENA-78/CXCL5 levels in the lungs and may affect angiogenesis and the inflammatory response in the airways of asthma patients.

## 1. Introduction

House-dust mites (HDM) (*Dermatophagoides pteronissinus*) allergens are among the most potent stimuli of asthma attacks [1, 2]. HDM excretions contain a plethora of biologically active compounds, which have allergenic potential that prompts IgE response in sensitized subjects. The same HDM compounds also have proinflammatory properties, which are independent of the IgE response. In asthma, neoangiogenesis of the airway wall is a recently recognized pathology, which contributes to airway wall thickness [3–5]. There is also evidence from animal models that HDM and other allergens can induce neovascularization of the inflamed airway [6, 7]. However, the effects of HDM allergens on asthma related angiogenesis remain incompletely understood.

HDM extracts have been shown to disrupt the tight junctions between epithelial cells and lead to the complete desquamation of the epithelial cell layer, hence facilitating the passage of allergens across the epithelial air-tissue border [8–10]. The major HDM allergen, Der* p1*, is a cysteine protease [11–13] and may be responsible for the observed epithelial cell desquamation, for the release of cytokines, and facilitates the transport of allergens across cultured epithelial cell layers [9–11, 14–16]. In consequence, the desquamation of the protective epithelium may allow allergenic compounds to penetrate deeper into the airway wall, thereby facilitating airway hyper-sensitization.

Proteases present in HDM excrements also exerted a direct modulatory effect on regulatory proteins in cultured human bronchial smooth muscle cells (BSMC) that affect the cell's ability to proliferate and to secrete cytokines [17]. In particular, BSMC exposed to HDM extract increased interleukin (IL)-6 production and cell proliferation through the protease-activated receptor 2 [17]. Hence, HDM exposure contributes to inflammation and airway wall remodeling in a mechanism independent of the immune system by direct interaction with BSMC. Furthermore, proteolytic activities present in fungal and bacterial extracts have been shown to specifically and dose-dependently degrade human interleukin (IL)-6 and IL-8/CXCL8 [18], as well as RANTES, monocyte chemotactic protein (MCP)-1, and epithelial-derived neutrophil attractant (ENA)-78/CXCL5 [19], hence reducing the bioavailability of these cytokines. Together, these data imply that the exposure of the airways to external factors with intrinsic proteolytic activity affects the relative level of immuno-modulatory cytokines, which may affect inflammation and microvessel formation and airway remodeling. In the present study, we analyzed the effect of HDM extract on the release and degradation of a panel of cytokines that have proinflammatory and angiogenic activity and are implicated in airway wall remodeling. Our data show that the proteolytic activity present in HDM extract degraded ENA-78/CXCL5 and reduced the migration of neutrophils. These findings imply that HDM allergens may have the capacity to modify the development of an eosinophil dominated inflammatory response in the lung.

## 2. Methods 

### 2.1. Isolation of Primary Bronchial Smooth Muscle Cells

BSMC were obtained from endobronchial biopsies obtained by flexible bronchoscopy or from therapeutically resected bronchial tissue obtained from the Department of Internal Medicine and Pneumology (University Hospital Basel, Basel, Switzerland) with the approval of the local ethical committee (EK: 05/06) and written informed consent of all patients. Patients with mild to moderate asthma had reversible airway obstruction documented in the past, with a median FEV1 of 70.5% of the predicted value (ranging from 45.3% to 84.6%). The samples used as nonasthmatic controls participating in this study were either from healthy organ donors whose lungs were deemed unfit for use in a transplant procedure or from the healthy part of lung resection material obtained after lung surgery.

Isolation of BSMC was performed as described earlier [20, 21]. BSMC bundles were dissected from the surrounding tissue by microscopy and pure muscle bundles were plated in a 25 cm^2^-flask prewetted with 1 mL of Dulbecco's modified Eagle's medium (DMEM) containing GlutaMax 4.5 g/L glucose (Gibco, Bioconcept, Allschwil, Switzerland), 5% FCS, 1x antibiotics-antimycotics, and 1x modified Eagle's medium vitamin mix (Invitrogen, Lubio, Luzern, Switzerland). BSMC were characterized by immunostaining as described earlier [22].

### 2.2. Generation of Conditioned Medium (CM)

BSMC were grown in BSMC-growth medium (GM): RPMI 1640 supplemented with 5% fetal calf serum (FCS), 8 mM L-glutamine, 20 mM hydroxyethyl piperazine ethane sulfonic acid, 1x antibiotics-antimycotics, and 1x modified Eagle's medium vitamin mix (Invitrogen, Lubio, Luzern, Switzerland) under normoxic conditions (21% O_2_, 5% CO_2_, and 37°C). BSMC were seeded and kept in BSMC-GM for 24 h before they were serum deprived for 24 h prior to stimulation. A stock solution (1 mg/mL) of HDM extract (gift from: Peter Adler Wurtzen, Alk Abello's Research Department, Hørsholm, Denmark) was prepared in RPMI 1640 medium and diluted in RPMI 1640 to the final concentration (20, 10, and 2 *μ*g/mL), which was then sterilized by filtration (0.22 mm) (MN Sterilizer PES; Macherey-Nagel AG, Oensingen, Switzerland). 10 ng/mL of colistin (Roth, Arlesheim, Switzerland) was to avoid effects of lipopolysaccharids (LPS) in HDM extract. To obtain HDM-CM, BSMC were incubated for 72 h (37°C) with HDM extract (2 and 10 *μ*g/mL) or without HDM extract in the presence or absence of 5% FCS.

### 2.3. Angiogenesis Antibody Array

The human angiogenesis antibody array G 1000 (Raybiotech, Lucerna-Chem, Luzern, Switzerland) was used to detect the release of 20 angiogenesis-related cytokines and growth factors in CM obtained from BSMC stimulated with HDM extract or unstimulated BSMC. 100 *μ*L of undiluted CM collected after 72 h was applied to each array and the angiogenesis related factors were evaluated according to the manufacturer instructions. Cy3 fluorescence was measured by a NimbleGen MS 200 microarray scanner (Roche, Basel, Switzerland) and signal intensity was analyzed by AIDA software (Raytest, Straubenhardt, Germany).

### 2.4. Cytokine-ELISA

Enzyme linked immunosorbent assay (ELISA) kits for IL-8/CXCL8 (sensitivity: 7.5 pg/mL), ENA-78/CXCL5 (sensitivity: 15 pg/mL), and VEGF (sensitivity: 9 pg/mL) were purchased from R & D (Abingdon, UK) and ELISA was performed according to the manufacturer's instructions. 100 *μ*L of CM obtained from BSMC was measured undiluted (VEGF), 1 : 5 diluted (ENA-78/CXCL5), or 1 : 50 diluted (IL-8/CXCL8).

### 2.5. ENA-78/CXCL5 mRNA Expression by RT-PCR

To induce mRNA, BSMC were grown in absence and presence of HDM extract (20 *μ*g/mL) for 6 h. Total RNA was extracted from 10^6^ cells (*t* = 0 h, and *t* = 6 h) by Trizol method (Gibco, Bioconcept, Allschwil, Switzerland). Then, 5 *μ*g of total RNA was converted into cDNA. Semiquantitative PCR (94°C, 65°C, and 72°C) was performed for 28 cycles using a Thermo Hybaid PCR Express (Catalys, Wallisellen, Switzerland) as described elsewhere [23]. The primers for ENA-78/CXCL5 were forward: 5′-ATC TCC GCT CCT CCA CCC AGT-3′ and reverse: 5′-TTC TTG TCT TCC CTG GGT-3′ generating a PCR fragment of 493 bp. The primers GAPDH were forward: 5′-CCA AAG GGT CAT CAT CTC TGC-3′ and GAPDH reverse: 5′-ATT TGG CAG GTT TTT CTA-3′ generating a PCR fragment of 417 bp.

### 2.6. Neutrophil Migration Assay

Polymorphonuclear cells (PMNC) isolation and neutrophil chemotaxis anticoagulated blood was obtained from the local blood bank (Blutspendezentrum, Basel, Switzerland) after written informed consent of all donors. PMNC were isolated using PolymorphprepTM separation medium as recommended by the manufacturer (Axis-Shield, Axonlab, Baden, Switzerland). Chemotaxis was assessed using 96-well Boyden chamber (Neuroprobe, Gaithersburg, USA) as previously described [24]. The lower compartments of the 96-well chamber were filled with either 100 ng/mL recombinant ENA-78/CXCL5, or 10 ng/mL recombinant ENA-78/CXCL5 (stimulated for 24 h with or without 10 *μ*g/mL HDM extract), or 10 *μ*g/mL HDM extract in RPMI 1640, or RPMI 1640 alone, and then covered with a 5 *μ*m pore-sized polycarbonate filter (Neuroprobe, Gaithersburg, USA). Next, 200 *μ*L of neutrophil suspension (2 × 10^6^ cells/mL) was added to the upper compartments. After 2 h of incubation (37°C, 5% CO_2_) the upper compartment was removed and the filter was fixed and stained with Differential Quik Staining Kit (Polyscience, Brunschwig, Basel, Switzerland). The membrane was scanned with a desktop scanner, and the intensity of spots was analyzed using Image J software.

### 2.7. Statistics

Cytokine data are presented as mean ± S.E.M. Migration analysis is shown as mean ± S.E.M. after densitometric image analysis (Image J software, National Institute of Mental Health, Bethesda, Maryland, USA). Unpaired Student's *t* test was performed and *P* values < 0.05 were considered significant.

## 3. Results

### 3.1. HDM Extract Downregulated ENA-78/CXCL5 Protein Levels

**Figure 1:**
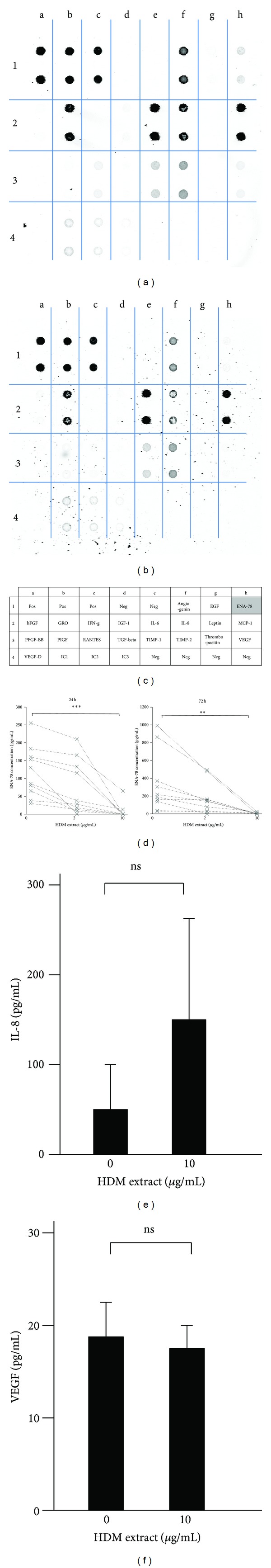
*Effect of HDM extract (HDME) on the release of angiogenic factors.* Typical examples of angiogenesis antibody arrays incubated with conditioned medium (CM) of unstimulated BSMC (a) and with CM of BSMC that were stimulated with 10 *μ*g/mL HDME (b). For identification purposes, the corresponding antibody array map is presented in (c). Angiogenic factors are identified by a letter-number code (e.g., IL-8/CXCL8 = f2, ENA78/CXCL5 = h1, VEGF = h3, etc.). Pos = positive control; Neg = negative control; IC1-IC3 = internal controls 1–3, and standard abbreviations are used for the detected angiogenic factors. (d) Time- and dose-dependent reduction of ENA-78/CXCL5 in the presence of CM of BSMC that were stimulated with 2 concentrations of HDM extract (*n* = 10) for either 24 or 72 hours. The same CM did not significantly affect IL-8/CXCL8 (e) and VEGF (f) levels. **P < 0.005; ***P < 0.001; ns = not significant.

To identify proteins that are affected by the addition of HDM extract to BSMC, CM of stimulated and unstimulated BSMC was applied to an antibody array. Comparing the protein expression patterns of 2 independent angiogenesis arrays, the expression of ENA-78/CXCL5 was consistently reduced in CM collected from BSMC that had been stimulated with HDM extract (Figures 1(a) and 1(b)). The panel of proangiogenic proteins of the array is shown in Figure 1(c). We then confirmed this finding with an ENA-78/CXCL5 ELISA in CM collected from BSMC stimulated with HDM extract for 24 and 72 h. As shown in Figure 1(d), HDM extract (10 *μ*g/mL) significantly decreased the concentration of ENA-78/CXCL5 in CM of stimulated BSMC (*P* < 0.05; *n* = 10). In contrast, using ELISA, we observed that HDM extract (10 *μ*g/mL) did not significantly alter the levels of IL-8/CXCL8 or VEGF protein (Figures 1(e) and 1(f)).

### 3.2. HDM Extract Did Not Affect ENA-78/CXCL5 mRNA Levels

**Figure 2:**
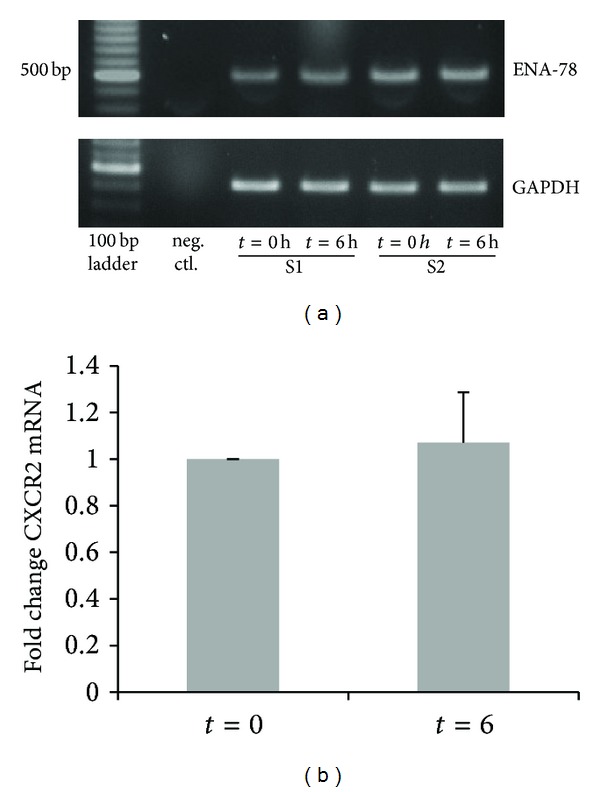
*Detection of ENA-78/CXCL5 mRNA by RT-PCR.* (a) Typical experiment showing ENA-78/CXCL5 (fragment size: 493 bp) and GAPDH (fragment size: 417 bp) RT-PCR products in BSM cells of 2 subjects (S1 = subject 1; S2 = subjects 2). (b) Densitometric analysis of RT-PCR as mean ± SEM. For details, see text.

To determine whether the HDM-dependent reduction of ENA-78/CXCL5 protein levels were associated with reduced gene transcription RT-PCR was performed using total RNA extracted after 0 and 6 h from BSMC grown in the absence and presence of HDM extract (20 *μ*g/mL). As shown in Figure 2, exposure to HDM extract did not significantly affect RNA levels of ENA-78/CXCL5.

### 3.3. HDM Extract Specifically Degraded ENA-78/CXCL5 Protein

**Figure 3:**
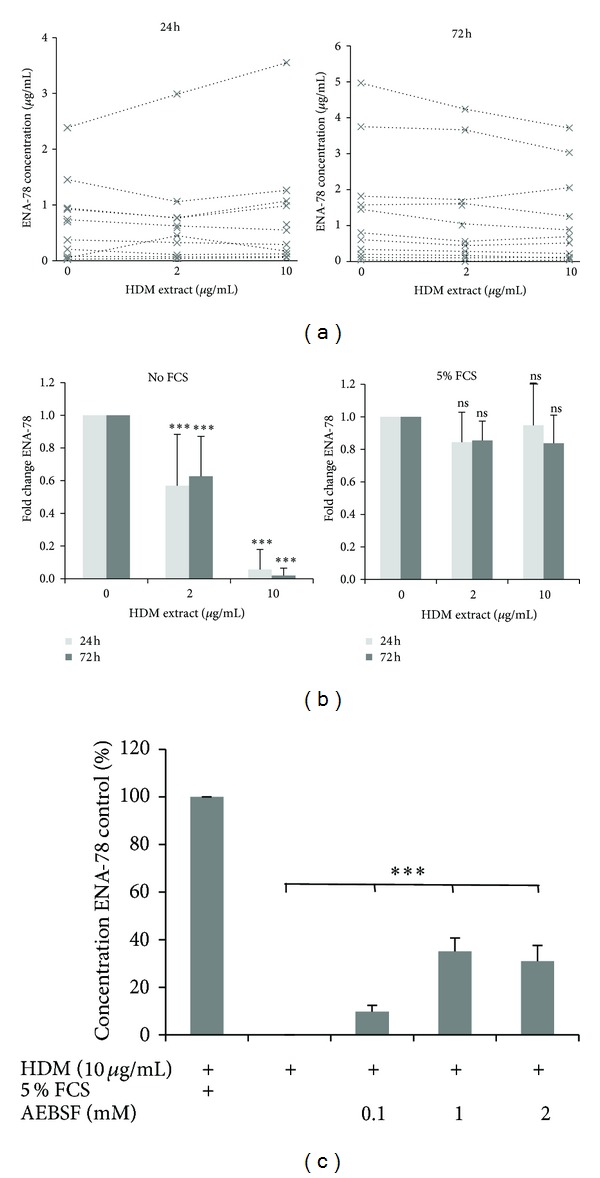
*FCS and AEBSF block the effect of HDM extract.* (a) Effect of HDM extract (2 and 10 *μ*g/mL) on ENA-78 levels in the presence of 5% FCS. ENA78/CXCL5 levels were measured in different HDM containing CM preparations after 24 h (left panel) and 72 h (right panel). (b) Effect of FCS (5%) on HDM extract-induced reduction of ENA-78/CXCL5 levels (*n* = 5; ***P < 0.001 comparing HDM extract stimulated CM to unstimulated CM). (c) Degradation of recombinant ENA-78/CXCL5 by HDM extract is inhibited by the addition of the serine proteases inhibitor AEBSF or FCS (*n* = 3, measured as duplicates; bars represent the mean ± SEM, ***P < 0.001 relative to the rescue effect of AEBSF on HDM extract-induced ENA-78/CXCL5 degradation).

The observation that HDM extract stimulation decreased ENA-78/CXCL5 protein levels while ENA-78/CXCL5 mRNA levels were unchanged indicated that the ENA-78/CXCL5 protein may be degraded by proteolytic activities of the HDM extract. Therefore, we incubated BSMC with 5% FCS which has strong antiproteolytic activity in the presence of HDM extract (2 and 10 *μ*g/mL). As shown in Figure 1(d), HDM extract reduced the ENA-78/CXCL5 protein level in CM collected from BSMC. In the presence of 5% FCS, this effect was completely abrogated (Figure 3(a)), suggesting a protease-dependent mechanism of ENA-78/CXCL5 degradation. Next, we tested the effect of HDM extract on the basal concentration of ENA-78/CXCL5 in CM from BSMC (*n* = 7) grown in the absence of FCS for 24 and 72 h. After collection of the CM, we incubated with either HDM extract alone or a combination of HDM extract + 5% FSC. In the absence of FCS, the addition of HDM extract significantly reduced the basal concentration of ENA-78/CXCL5 in CM. FCS abrogated this effect (Figure 3(b)). We confirmed the proteolytic activity of HDM extract and the antiproteolytic effect of FCS on ENA-78/CXCL5 degradation with recombinant ENA-78/CXCL5. Furthermore, we assessed the effect of the serine protease inhibitor AEBSF and the cysteine protease inhibitor E64 on the degradation of recombinant ENA-78/CXCL5 by HDM extract. Incubation with E64 (0.5 *μ*M, 5 *μ*M, and 10 *μ*M) did not prevent ENA-78/CXCL5 degradation by HDM extract in any of the used concentrations (data not shown). In contrast, the addition of AEBSF led to a significant increase in ENA-78/CXCL5 concentration compared to control condition (without AEBSF) (Figure 3(c)).

### 3.4. HDM-CM Reduced Chemotaxis of Neutrophils

**Figure 4:**
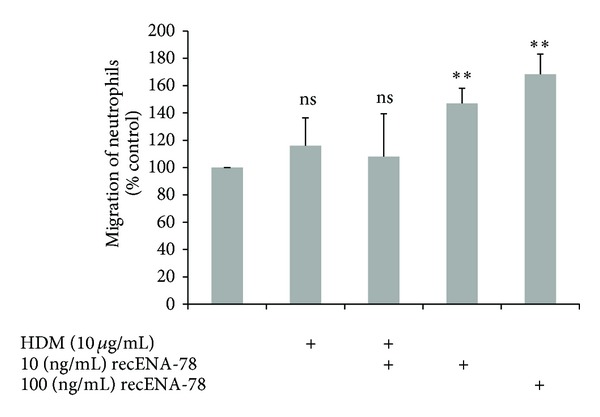
*HDM extract reduces recombinant ENA-78/CXCL5-induced neutrophil migration.* Migrated cells were stained with Diff Quick staining kit, scanned with a desktop scanner and densitometric analysis was performed with Image J. Values are expressed as mean ± SEM. **P < 0.005; *n* = 3 (neutrophils from 1 healthy subject in triplicate).

Finally, we tested the effect of HDM extract-dependent ENA-78/CXCL5 degradation in regard to the chemotactic capacity of ENA-78/CXCL5. As shown in Figure 4, ENA-78/CXCL5 alone dose-dependently induced neutrophil migration compared to medium only, while HDM extract alone had no significant effect on the induction of migration when combined HDM extract reduced the neutrophil migratory effect of ENA-78/CXCL5.

## 4. Discussion

In our study, we demonstrated for the first time that the CXCR2 ligand ENA-78/CXCL5 was degraded upon exposure to HDM extract, whereas a second CXCR2 ligand IL-8/CXCL8 was not affected. CXCR2 ligands have been suggested to play a crucial role in inflammation and angiogenesis, in particular in directing neovascularization in tumors [25]. We recently showed that angiogenic CXCR2 ligands are produced by human BSMC directing sprout outgrowth from endothelial cell spheroids [unpublished data, manuscript submitted]. In addition to its angiogenic characteristics, ENA-78/CXCL5 exerts strong chemotactic properties for neutrophils [26]. Several neutrophil attracting chemokines are present in the lung, including IL-8/CXCL8, growth related proteins (GRO/CXCL1-*α*, *β*, *γ*), and ENA-78/CXCL5 [27, 28]. Although ENA-78/CXCL5 and IL-8/CXCL8 share some properties, each cytokine has its own specific effects [29–33]. Comparing these studies, ENA-78/CXCL5 seems to be more important in chronic than in acute immunological responses.

The observation that FCS completely blocked the degradation of ENA-78/CXCL5 protein demonstrated that the degradation was due to the proteolytic activity present in the HDM extract and this was confirmed by the protective effect of the serine protease inhibitor AEBSF. In contrast, the cysteine protease inhibitor E64 did not prevent HDM extract-dependent ENA-78/CXCL5 degradation; therefore, we conclude that one mechanism of ENA-78/CXCL5 degradation is dependent on serine proteases present in HDM extract. Proteolytic activities present in HDM extracts have been extensively studied and several HDM allergens have been identified as proteases [34]. A major effect of these proteases is the selective reduction of the bioavailability of cytokines, since not all cytokines are equally prone to proteolytic degradation [19]. Diminished bioavailability of cytokines due to proteolytic degradation has been associated with opportunistic fungi, in particular* Aspergillus fumigatus* [18], as well as with pathogenic bacteria, such as* Pseudomonas aeruginosa* [19]. Characteristic for both organisms is the secretion of a wide range of proteolytic enzymes intended for growth and existence. Present in the lung, these enzymes may help the microorganisms to penetrate into deeper areas of the airway wall. In addition, their protease-activity may change the relative quantities of cytokines of the airways and skew the immune system. Proteolytic modifications of ENA-78/CXCL5 protein have been shown to reduce and to enhance its biological activity. Cleavage of ENA-78/CXCL5 by MMP, MMP2, or the aminopeptidase CD13 has been shown to enhance its activity [35, 36], whereas proteolytic modifications caused by metalloproteases from* Pseudomonas aeruginosa* were accompanied by the loss of chemotactic activity [19]. We were able to provide evidence that proteolytic modifications by HDM extract abrogate the neutrophil-attracting properties of ENA-78/CXCL5.

Our data provide evidence that HDM proteases may directly affect the proinflammatory and proangiogenic cytokine response through proteolytic degradation of ENA-78/CXCL5 protein. Produced by epithelial cells, ENA-78/CXCL5 was first described as a chemoattractant for neutrophils [31]. We have recently reported that not only epithelial cells but also BSMC release high levels of ENA-78/CXCL8 [37]. Continuous exposure to HDM allergens may reduce the bioavailability of ENA-78/CXCL5 due to proteolytic degradation and lower the number of neutrophils that infiltrate the asthmatic airway. This may alter the immune response in favor of eosinophilic inflammation, as it is often observed in the lungs of asthmatic patients and thought of as the main histopathology of the asthmatic lung [38–40]. It should be noted, however, that asthma sometimes is associated with neutrophilia. In a sheep model of asthma, HDM extracts induced neutrophil infiltration at 6 h, followed by eosinophils and activated lymphocytes into the lung tissue and BAL, similar to the late-phase allergic response seen in human asthma [41]. It is currently unknown whether or not an association exists between exposure to HDM allergens and eosinophilia. However, selective degradation of ENA-78/CXCL5 by proteases present in HDM extract might provide a partial explanation.

Our experiments with the Boyden chamber showed that chemotactic migration of neutrophils towards ENA-78/CXCL5 is reduced in the presence of HDM extract. The microenvironment–such as the presence of serum (e.g., due to increased vascular leakage), as well as the relative presence of cytokines and chemotactic factors–will ultimately determine the immunological response. A common pathological feature of inflammatory airway diseases is an imbalance of proteases and antiproteases present in the lining fluid of the lungs. The presence of serum-derived antiproteases may further delineate a balanced equilibrium between proteases and antiproteases, as bystander effects neutralize the protease-activity of inhaled compounds. An external addition of foreign proteases, which may be derived from fungi, bacteria, or HDM, may distort this intricate balance and lead to irreversible impairment of airway function and disease.

Taken together, our data demonstrate that the ENA-78/CXCL5 protein is susceptible to degradation by proteases present in HDM extract. Although reduced bioavailability of ENA-78/CXCL5 could potentially cause atypical immunological characteristics* in vivo*, the biological relevance of this observation remains to be established.
